# Clinical Aspects and Molecular Mechanisms of Cognitive Dysfunction in Children and Adolescents with Type 1 Diabetes

**DOI:** 10.3390/children13030416

**Published:** 2026-03-18

**Authors:** Eleni Angelopoulou, Nicolas C. Nicolaides, Alexandros Gryparis, Tania Siahanidou, Panagiota Pervanidou, Christina Kanaka-Gantenbein

**Affiliations:** 1 Diabetes Center, Division of Endocrinology, Diabetes and Metabolism, First Department of Pediatrics, Medical School, National and Kapodistrian University of Athens, “Aghia Sophia” Children’s Hospital, 11527 Athens, Greece; elenangel@med.uoa.gr; 2 Medical School, University of Cyprus, 1678 Nicosia, Cyprus; nicolaides.nicolas@ucy.ac.cy; 3 Department of Speech and Language Therapy, University of Ioannina, 45500 Ioannina, Greece; alexandros@uoi.gr; 4 Neonatology Unit, First Department of Pediatrics, Medical School, National and Kapodistrian University of Athens, “Aghia Sophia” Children’s Hospital, 11527 Athens, Greece; siahan@med.uoa.gr; 5 Unit of Developmental and Behavioral Paediatrics, First Department of Pediatrics, National and Kapodistrian University of Athens, “Aghia Sophia” Children’s Hospital, 11527 Athens, Greece; ppervanid@med.uoa.gr

**Keywords:** type 1 diabetes, cognitive dysfunction, diabetic ketoacidosis, early diabetes onset, diabetes complications, oxidative stress, hypoglycemia, glucose fluctuations

## Abstract

Type 1 diabetes (T1D) constitutes a chronic metabolic disorder attributed to the autoimmune destruction of insulin-producing pancreatic β cells, which most frequently occurs in childhood. Long-term complications of T1D are expected to occur mainly in adult life, whereas cognitive dysfunction can also occur in children and adolescents with T1D. Most studies demonstrate mild cognitive impairment, especially in the domains of memory, attention and executive functions, all of which affect academic performance, which may also negatively influence adherence to appropriate glucose monitoring and insulin treatment in children and adolescents with T1D. As a result, mild cognitive dysfunction can be an obstacle to both optimal glycemic control during childhood and adolescence and academic achievements for young individuals with T1D. The major metabolic changes occurring around the onset of diabetes, such as severe hyperglycemia and diabetic ketoacidosis, may have a negative impact on brain plasticity during this vulnerable period of neurodevelopment, especially in children diagnosed at a younger age. The pathophysiological mechanisms involved are closely related to increased oxidative stress and the accumulation of advanced glycation end products in the brain, thus leading to neuron cell damage and apoptosis. On the other hand, hypoglycemic episodes and glucose fluctuations may also impair neuronal integrity. The aim of the current narrative review is therefore to present the existing literature data on the clinical aspects, risk factors and molecular mechanisms associated with cognitive dysfunction in children and adolescents with T1D.

## 1. Introduction

Type 1 diabetes (T1D) is the most common type of diabetes in childhood and constitutes a chronic metabolic disorder occurring after the autoimmune destruction of the insulin-producing pancreatic beta cells, leading to hyperglycemia and finally diabetic ketoacidosis if diagnosis is delayed [1]. The biological processes leading to this autoimmune destruction are not precisely identified, although it is widely accepted that when a genetically predisposed individual is exposed to environmental factors such as viral or bacterial infections, of which several are still undefined, an inappropriate immunological response, specifically against pancreatic β-cells, takes place, leading to progressive β-cell destruction and ultimately leading to reduced insulin production [2,3,4,5]. Upon diagnosis, children and adolescents with T1D are treated with exogenous insulin administration and, along with their families, they are guided, educated and empowered by a multidisciplinary team of health care professionals, the diabetes team, to undertake all necessary steps, including glucose monitoring, carbohydrate counting, insulin administration, and sick-day and sports management, to face all possible challenges related to diabetes [6].

The aim of T1D treatment is to achieve optimal glycemic control, from the onset of the disease, to avoid and eliminate acute and chronic diabetes complications [7,8]. The introduction of new technologies in T1D management, such as continuous glucose monitoring (CGM) and continuous subcutaneous insulin infusion (CSII), otherwise called insulin pump therapy and advanced hybrid closed-loop systems (AHCL), has significantly contributed to improved quality of life and better glycemic control, reducing the diabetes burden and minimizing the risk of acute complications, as well as the time spent in hypo- or hyperglycemia [9,10]. Adult patients with T1D are commonly expected to face long-term complications, including microvascular complications such as diabetic retinopathy, nephropathy, and neuropathy, as well as macrovascular complications if long-term hyperglycemia persists over the years. The recommended screening for early detection of these complications begins during childhood and adolescence, or 2–5 years after diabetes onset [11].

Besides the well-known acute complications, such as hypoglycemic events or diabetic ketoacidosis (DKA), and the afore-mentioned long-term complications of micro- and macrovascular origin, T1D is also correlated with cognitive impairment, which constitutes another, although less identified, complication of the disease, which can even occur during childhood. The prevalence of cognitive impairment in children with T1D is difficult to estimate, firstly due to its insidious course and lack of established diagnostic tools, and secondly due to the variety of cognitive assessment tools used in different studies. Most investigators describe the statistically significant lower scores of children with T1D compared to healthy controls without referring to the prevalence of the complication. Among adult patients with T1D or type 2 diabetes (T2D), the risk of developing dementia is estimated to be 40–60% higher than in healthy controls and adult patients with T2D are at a 20% higher risk of developing mild cognitive impairment [12]. Interestingly, even a mild decline in the cognitive function of children and adolescents with T1D can result in poorer adherence to the appropriate glucose monitoring and insulin treatment, thus leading to inadequate glycemic control, further aggravating long-term prognosis [13]. Specifically, diabetes onset at a very young age constitutes a major risk factor for cognitive dysfunction, affecting the school performance, neurodevelopmental outcome or even adherence to treatment of these young people. Factors related to diabetes per se, such as metabolic changes occurring close to diabetes diagnosis, age of onset, diabetes duration and chronic inflammation and the subsequent macro- or microvascular dysfunction, can all further contribute to the development of cognitive impairment in patients with T1D [14]. The aim of the current narrative review is therefore to present the existing literature data for the clinical aspects, risk factors and molecular mechanisms associated with cognitive dysfunction in children and adolescents with T1D.

## 2. Materials and Methods

A literature search was conducted from 1996 onwards by searching peer-reviewed publications in the PubMed electronic database, using the terms “children”, “type 1 diabetes”, “cognitive dysfunction”, “diabetic ketoacidosis”, “hyperglycemia, “hypoglycemia”, “age at diabetes onset”, “brain damage”, “molecular mechanisms”, and “biomarker”. Inclusion criteria included: children and adolescents up to 20 years old with T1D, reference to cognitive dysfunction in children with T1D, responsible risk factors, molecular mechanisms, and possible biomarkers of brain damage in diabetes. Only original research articles, clinical practice consensus guidelines, or reviews written in the English language were considered. Exclusion criteria included studies concerning cognitive dysfunction in adults without examining risk factors in childhood. Studies concerning molecular mechanisms and biomarkers conducted in adults were not excluded, due to the conditions of inflammation underlying both T1D and T2D and due to the research gap in children with T1D. Appendix A presents the conducted risk of bias assessment of the selected studies.

## 3. Clinical Aspects of Cognitive Dysfunction in Children with T1D

### 3.1. Domains of Cognitive Dysfunction in Children with T1D

Compared to healthy age-matched controls, children and adolescents with T1D have lower performance in various cognitive domains, as supported by the extensive literature data, comprising data from well-designed studies during recent decades [15,16,17,18,19,20,21,22,23,24]. As Dahlquist et al. have shown, school performance in a large cohort of children with T1D was lower than that of age-matched controls without T1D, especially in topics such as Sports, English, Swedish and Mathematics, and this finding seemed to be more significant in children of a younger age at diabetes onset and consequently larger diabetes duration [15]. Many studies also focus on the assessment of memory [16,17,18], attention [19,20] and executive functions [21,22,23], revealing cognitive deficits in children with T1D when comparing them with healthy age-matched controls; this occurs due to further aggravating factors, such as the negative impact of long periods of hyperglycemia and one or more episodes of diabetic ketoacidosis, either at diagnosis or across the lifespan of a child with diabetes. Overall, memory, intelligence quotient, psychomotor efficiency, executive functions, visual attention, visual perception, and cognitive flexibility are the main neurocognitive functions that appear to be affected [24].

### 3.2. Neuroimaging Findings Related to Cognitive Dysfunction in Children with T1D

Neuroimaging findings based on functional magnetic resonance imaging (fMRI) have demonstrated that neuronal damage and myelinated fibrous neuronal damage are associated with lower performance of children with T1D on cognitive testing [25,26]. When investigating the impact of DKA on brain structure, firstly within 48 h after diabetes diagnosis and in three time points post-diagnosis, i.e., 5 days, 28 days, and 6 months after diabetes onset, alterations, especially in white matter volume but also in gray matter volume and N-acetylaspartate, have been described [27]. Interestingly, children who experienced DKA without having clinical signs of cerebral edema exhibited an increase in white matter volume at the time of diagnosis, as well as lower levels of NAA, which could indicate neuronal damage at the molecular level and dysfunction. Although these structural changes were in many cases no longer visible 6 months later, the memory and attention deficits of those children persisted at the time of the 6-month follow-up, suggesting the long term, irreversible effect of DKA on the cognitive function of children with T1D and the clinical significance of metabolic memory, i.e., the continuous negative impact of prior exposure to high blood glucose levels at the molecular level, even after a child with T1D reaches optimal glucose control, thus maintaining the risk of micro- and macrovascular diabetes complications. Furthermore, except for those alterations taking place at the time of T1D diagnosis, structural changes in white and gray matter volume were described to be present 6 years after T1D diagnosis and to persist up to 12 years post-diagnosis in the 6 years of follow-up in the longitudinal study of Mauras et al. These alterations were associated with worse glycemic control across the follow-up period [28], underlining the negative impact of hyperglycemia. Furthermore, using the advanced neuroimaging technology of diffusion tensor imaging (DTI), which can detect the mobility of water molecules through the white matter, thus revealing impairment of axonal organization, connection and communication in different areas of the brain, Toprak et al. [29] have revealed alterations in neuronal pruning and organization at different brain regions in children diagnosed with T1D: findings that were also correlated, at the time of the examination, with altered performance in cognitive tasks evaluating verbal intelligence or memory in comparison to age-matched healthy controls. Finally, the recent study of O’Connell et al. has revealed the negative effect of acute hyper- and hypoglycemia on brain perfusion using fMRI, which persisted for at least 45 min after recovery to euglycemia in adolescents with T1D [30].

The above neuroimaging findings emphasize the negative impact of even a single episode on white matter structure and neuronal function at the time of T1D diagnosis, but also the acute and longitudinal effect of inadequate glycemic control on brain structure that can be linked to cognitive dysfunction in patients with T1D, which can either manifest close to diabetes diagnosis or later, during childhood and adult life.

### 3.3. Main Risk Factors for Cognitive Impairment in Children with T1D

#### 3.3.1. Diabetic Ketoacidosis

Diabetic ketoacidosis (DKA) is a serious, potentially life-threatening, acute complication of diabetes mellitus that predominantly supervenes at diabetes onset, although it can also occur any time during the life course of a patient with T1D [10]. Morphological and functional brain changes in magnetic resonance imaging (MRI) and spectroscopy, as well as lower performance in cognitive testing for memory, attention and mental state, have been described in children with T1D who experienced DKA at diabetes diagnosis compared to children with T1D who did not [27]. DKA, as a major metabolic imbalance state due to absolute lack of insulin and consequent severe hyperglycemia on the one hand and severe oxidation state on the other, can cause severe brain damage both at the time of DKA as well as in the long term, leading to cognitive decline already being present in childhood [31,32]. As Semenkovich et al. have shown, deficits in spatial memory are present even 3.5 months after diabetes onset in children who have experienced DKA compared to those who were diagnosed with hyperglycemia without DKA [18]. Moreover, not only the presence but also the severity of DKA at diabetes onset seems to play a major role [16,32]. Subtle deficits in memory as well as lower intelligence quotient (IQ) scores have been observed in a large cohort of children with T1D who were assessed after 2–6 months after experiencing a DKA episode [16]. Interestingly, in this prospective randomized multicenter clinical study, Ghetti et al. have described that among 758 children with T1D, aged 6–18 years, with at least one DKA episode at diagnosis or after diabetes onset, those who experienced moderate or severe DKA had lower IQ and memory task scores (item-color recall and forward digit span) compared to those with a mild episode of DKA [16]. Among the newly diagnosed children, the presence of moderate/severe DKA negatively influenced the item-color recall, indicating impairment in memory function that is already present shortly after diabetes diagnosis. Regarding preschool children, 46 children with T1D, aged 3–5 years old, with DKA at the disease onset, were assessed for their cognitive function at 2–6 months after DKA and were compared to 27 children with T1D, matched for age and diabetes duration, without DKA at diagnosis [33]. Preschool children with a history of DKA at diagnosis exhibited lower IQ scores compared to those without DKA, indicating the negative impact of DKA in preschool children, independently from the severity of the DKA episode, as the severity of DKA in this age group seemed to have no influence on the described cognitive impairment. The executive function of children with T1D can also be impaired by DKA episodes. A prospective study evaluated the cognitive executive function of a cohort of 99 children and adolescents with T1D and a history of DKA: either one episode of DKA at diagnosis (*n* = 89) or two episodes of DKA (*n* = 7), three episodes of DKA (*n* = 2), five episodes of DKA (*n* = 1) during the patient’s lifetime. There were 82 children with T1D without a history of DKA and 100 healthy age-matched controls, all aged 7–18 years old, using the Wisconsin Card Sorting Test (WCST) [21]. The DKA group recorded a higher rate of errors in the WCST, compared with the healthy controls, as well as a lower mean IQ score. Importantly, the authors reported that these differences were associated with the age of the children when they experienced a DKA episode. Neither of the above-mentioned differences in executive function and IQ score were noted between the healthy controls and the group of children with T1D without DKA, confirming the major contribution of DKA as an important risk factor for cognitive impairment in children with T1D.

Consequently, based on large recent longitudinal studies, the presence of DKA, especially at diabetes diagnosis, as this timepoint is mainly investigated, rather than DKA episodes during the child’s lifetime, seems to play a critical negative role on memory, intelligence and attention among children with T1D who experience DKA at diagnosis. Furthermore, the severity of the DKA episode seems to be an additional contributing factor, resulting in even more profound cognitive impairment when a moderate or severe rather than a mild DKA episode occurs. A lack of control group, different cognitive assessment tools and socio-economic factors in each study could explain the discrepancy of the different results.

Table 1 summarizes studies presenting DKA as a risk factor playing a role in cognitive impairment in children with T1D.

#### 3.3.2. Age at Diabetes Onset

As mentioned above, children’s age at T1D onset is another determinant of neurocognitive impairment [13]. In recent decades, an increase in the number of children diagnosed with T1D under the age of six has been observed worldwide [39,40]. Early childhood constitutes a vulnerable period of human growth, due to crucial changes taking place in brain growth and development, including neuronal synapsis, function and structure [41]. As a result, the brain’s exposure to metabolic derangements provoked by severe hyperglycemia or DKA, more frequently observed in this age group, can lead to brain damage and, consequently, neurocognitive impairment [34,35,42,43,44,45]. Mazaika et al. have shown that young children with T1D demonstrate structural changes in total gray and white matter growth when compared with healthy controls, and this effect is age-dependent, as more devastating changes are observed in the age groups under 7 years old [42]. In addition, children diagnosed with T1D under the age of 5 years had an increased risk of learning difficulties when compared with healthy controls, which also influenced their subsequent academic skills. Interestingly, this correlation was irrelevant to the history of DKA or severe hypoglycemia, highlighting the importance of the age at diagnosis per se as an important risk factor for future cognitive decline [34]. Regarding the influence of the early onset of the disease in the cognitive domains of memory, attention and executive function literature data is contradictory, probably due to different sample populations (children, adolescents, early or late adulthood), sample sizes and methods used in each study for cognitive assessment [35,43,44,45,46,47]. Overall, well-structured studies indicate a younger age at diabetes onset as being a risk factor for the neurocognitive development of these patients, negatively influencing their scoring in intelligence, attention and executive function testing compared to the healthy controls. In addition, longitudinal alterations in brain structure have also been linked to a younger age at T1D diagnosis, further indicating the negative impact of early-onset T1D on brain development and neuronal structure.

Table 2 summarizes some of the studies suggesting the age of T1D onset as a possible risk factor for cognitive impairment in children with T1D.

#### 3.3.3. Hyperglycemia

The negative impact of hyperglycemia on the cognitive function of children with T1D was already recognized at least two decades ago [48]. Acute hyperglycemia was shown to be responsible for the lower performance on the Wechsler Intelligence Scale for Children (WISC) of 12 children with T1D and blood glucose above 20 mmol/L compared to their performance in the same test at a time of euglycemia (blood glucose below 10 mmol/L) [49]. Moreover, similar results were described in a recent study by Suput Omladič et al., where the spatial working memory of twenty adolescents with T1D, evaluated by brain activation in specific brain regions through functional MRI (fMRI), has been negatively affected by acute hyperglycemia (blood glucose above 20 mmol/L) at the time of testing: an important finding, considering that such memory deficits can consequently influence academic performance, daily activities and compliance to glucose monitoring and insulin treatment [17]. Chronic, apart from acute, hyperglycemia is associated with differences in white matter microstructure, as is shown in the recent longitudinal study by Mauras et al., where poor glycemic control was associated with lower fractional anisotropy, indicating lower white matter integrity and myelination throughout the years of follow-up [50]. Large periods of exposure to high blood glucose levels can lead to neurocognitive dysfunction, influencing domains such as school and academic performance [51], memory, verbal intelligence and attention [36,37,52]. Interestingly, it has also been shown that lower performance in psychomotor speed processing at the time of diagnosis, independently of the presence of DKA, can predict poorer glycemic control even one year after T1D diagnosis, highlighting the bilateral relationship between glycemic control and cognitive function [38]. Finally, long periods of hyperglycemia, leading to higher HbA1c, have a negative impact on cognitive function appearing in childhood and persisting into adulthood [53].

Finally, not only acute but also chronic hyperglycemia can be responsible for cognitive impairment in children with T1D, presenting even in the first year after diagnosis, when optimal glycemic control cannot be achieved. Data from recently published neuroimaging studies depicted the alterations in brain volume and structure that accompany hyperglycemia and lower scoring in cognitive tests for those children [28,30].

Table 3 shows studies presenting hyperglycemia as an important risk factor for cognitive impairment in children with T1D.

#### 3.3.4. Hypoglycemia

Recurrent episodes of hypoglycemia and episodes of severe hypoglycemia have been described as factors influencing cognitive function in children and adults with T1D, mainly due either to neuronal destruction or impaired neuronal pruning [54,55,56]. Though hypoglycemia’s critical role on cognitive impairment in patients with diabetes remains controversial in the international literature [13], severe hypoglycemia is undoubtedly positively linked to brain and neuronal damage and dementia in older people with either type 1 or type 2 diabetes [57,58,59].

The introduction of intensive therapies in T1D three decades ago, aiming at eliminating long-term diabetes’ microvascular complications, has raised a global concern that longer time spent in hypoglycemia in patients with T1D may probably lead to a negative impact on their brain function. Interestingly, in the SDIS study and the DCCT study, a large cohort of adults with T1D who were treated with intensive insulin treatment and experienced higher rates of hypoglycemia exhibited no difference in intelligence and memory testing in comparison to those under conventional treatment when assessed after 10 and 18 years of follow-up, respectively [60,61]. Similarly, in the results from the subgroup of adolescents from the DCCT study who have been followed up for the same period of 18 years, no correlation was revealed between hypoglycemia and cognitive decline [62]. Nevertheless, many studies over the last decades underline the negative effect of hypoglycemia on brain function and the domains of learning, attention and memory [63,64,65]. However, intensified insulin treatment at the era of the DCCT study mainly comprised a multiple daily injections (MDI) regimen and not the application of the newer continuous subcutaneous insulin infusion (CSII)/insulin pump regimens. Currently, the wide application of automated closed-loop systems in T1D treatment in children worldwide is expected to contribute to the improvement of glycemic control while minimizing or even eliminating episodes of severe hypoglycemia, thus preventing the deleterious effects of severe hypoglycemic episodes on cognitive function, even though recent data show that a large proportion of patients with T1D still experience severe hypoglycemia [66]. In addition, Verhulst et al. have recently shown that severe hypoglycemic events can cause brain damage, regardless of the presence of diabetes or hypoglycemia awareness. Ninety-four adults were recruited in this study: 26 patients with T1D and hypoglycemia awareness; 21 patients with T1D and hypoglycemia unawareness; 15 patients with type 2 diabetes under insulin treatment; and 32 healthy controls, who were age-, sex- and BMI- matched with either the T1D group (16 participants) or type 2 diabetes group (16 participants) [67]. According to the results of this study, an episode of acute hypoglycemia (cut off level < 3 mmol/L) could increase the participants’ reaction time in specific cognitive tests, with this effect being consistent in the different subgroups examined. In addition, the participants’ performance in memory tasks was lower in circumstances of hypoglycemia, irrespective of the presence of diabetes, the type of diabetes and the level of hypoglycemia awareness. These findings indicate the negative impact of clinically significant hypoglycemia on cognitive function, regardless of the presence of other clinical factors. Consequently, it is of great importance to take advantage of the available diabetes technology for glucose monitoring and insulin treatment in order to achieve glycemic targets, thus eliminating the time spent in hyperglycemia, which is undoubtedly harmful for brain development, without at the same time increasing the risk of cognitive impairment due to hypoglycemia, as has been demonstrated in large peer-reviewed longitudinal studies.

Table 4 presents the most relevant studies investigating the role of hypoglycemia on the cognitive function of patients with T1D.

#### 3.3.5. Glucose Variability

Glucose variability, which can be easily assessed by the continuous glucose monitoring systems that people with T1D use, is another measure for the assessment of the quality of glycemic control. Glycemic variability can exaggerate the oxidative stress that already takes place in people with diabetes, thus leading to further endothelial dysfunction and direct neuronal damage associated with cognitive impairment, as demonstrated in animal models and cell lines [68]. Interestingly, 8 iso PGF2a, a marker of oxidative stress, has been associated with glucose variability, though the results are controversial among studies including people with either T1D or T2D. A recent review from Wang et al. has described the impact of glucose level fluctuations on various diabetes-related conditions such as neuropsychiatric disorders and liver and bone disease [69]. Studies including mainly people with T2D have demonstrated a correlation between extreme fluctuations in glucose levels and the risk for neurocognitive impairment and Alzheimer’s disease [70,71]. Studies including adults with T1D are few, and there are even fewer in children and adolescents [42,54], though they indicate the role of glycemic excursions, especially in processing speed and visuospatial task performance, as glucose is the main energy fuel for the brain and neurons are dependent on the extracellular glucose concentration without having the ability to adjust to changes in blood glucose [72]. The study of Meng et al. has shown a significant positive correlation of urine 8-iso-PGF2a/Cr with indicators of glucose variability, such as MBG, SDBG, MAGE, and IAUC in children with T1D. Interestingly, this association was stronger as the disease progressed from diagnosis to 5 years post-diagnosis [73].

The above findings provide strong evidence that not only hyperglycemia or hypoglycemia but also the extended glucose variability itself can be responsible for disturbing the oxidative balance. Thus, further research regarding the correlation between continuous glucose metrics, such as the coefficient of variation, but also time in range (TIR), time above range (TAR) or time below range (TBR) with cognitive function is needed. Especially in children with T1D who widely use, upon diagnosis, the continuous glucose monitoring systems, such a correlation would be helpful to investigate the possible link between indicators of oxidative stress and glucose variability alone or together with metrics of hyper- or hypoglycemia.

### 3.4. Effect of New Therapy Technologies

Lately, new technologies, such as continuous glucose monitoring, sensor-augmented insulin pumps and closed-loop systems, as mentioned above, are widely used by people with T1D, and their use has led to improved glycemic control and better achievement of glycemic targets [74,75,76]. At the same time, their contribution to the quality of life of individuals with T1D and their families is of great importance [77]. Recent studies have additionally shown the positive impact of the use of the closed-loop systems on the cognitive function of adolescents with T1D, after 6 months of use, compared with adolescents using standard care practices (multiple daily injections or open-loop pumps). Especially, adolescents with T1D aged 14–18 years old exhibited improvement in their glycemic control, with less time spent in hyperglycemia and fewer glycemic excursions when using a closed-loop system for 6 months compared to those with standard diabetes insulin therapy. These findings were associated with differences in fMRI findings and higher IQ scores, indicating an improvement in cognitive function when achieving better glycemic control and minimizing glucose excursions [78].

## 4. Molecular Mechanisms

### 4.1. The Impact of Hyperglycemia at the Cellular Level

The brain is mainly dependent on glucose levels as its main energy source. The continuous function of neuronal cells requires a large proportion of the overall glucose uptake of the organism to achieve the appropriate performance of the different neurocognitive processes. Consequently, glycemic excursions, such as hypoglycemia and hyperglycemia, frequently observed in persons with any type of diabetes, can cause brain damage, leading to deficits in various cognitive domains [79]. A recent review from Yu et al. analyzes the main pathophysiological processes that are responsible for mild cognitive decline in persons with any type of diabetes. The role of chronic hyperglycemia is critical, especially in individuals with poor glycemic control, which leads to increased inflammation and oxidative stress, which also affect the central nervous system and induce neuroinflammation [80]. Hyperglycemia is a major risk factor for diabetes complications. Under conditions of high blood glucose levels, the increased production of advanced glycation end products (AGEs) results in the intravascular accumulation of these molecules, ultimately leading to microvascular complications of the disease. Since AGEs bind to macrophage receptors, such as RAGE (receptor for advanced glycation end products) and to macrophage scavenger receptors (MSR), resulting in the release of proinflammatory cytokines, this binding contributes to inflammatory processes in the periphery as well as in the nervous system, which can in turn induce neuronal death and apoptosis [81]. Other metabolic pathways that are disrupted in neuronal cells under hyperglycemic conditions are the pathway of polyoles, where large amounts of sorbitol are produced, resulting in a reduction in Na-K-ATPase, influencing synaptic communication, and the protein kinase C pathway, which is linked to diabetes’ microvascular complications and is activated under high blood glucose concentrations. All these alterations at the cellular level can aggravate the oxidative imbalance through the production of reactive oxygen species (ROS), thus increasing the oxidative stress and chronic neuroinflammation [82,83]. As the recent systematic review of Bornemann et al. has reported, chronic inflammation and oxidative stress seem to be major risk factors for the development of Alzheimer’s disease (AD) and neuro-senescence. Several proteins, such as complement components and chemokines, which are implicated in inflammatory processes, have been indicated as possible biomarkers for the early diagnosis of the disease, even at the presymptomatic stage [84]. Hippocampus, a brain structure with an important role in memory and learning, seems to be vulnerable to the conditions of oxidative stress. Thus, several studies in animal models have demonstrated that neuronal apoptosis and morphological changes, such as larger roughness and volume of hippocampal tissue, can occur under chronic inflammation and oxidative stress, negatively impacting its function [85]. In addition, less growth of the hippocampus has been described among children with T1D during an 18-month follow-up period in those with poor glycemic control, suggesting the negative role of hyperglycemia and great glucose excursions on the structure of the hippocampus [86]. It is evident that there is a need for further studies aiming to unravel the mechanisms that are responsible for neurocognitive impairment at younger ages, especially in children and adolescents. Apparently, such mechanisms are not linked to aging but to inflammation, especially neuroinflammation and oxidative stress affecting neurodevelopment in childhood and provoking mild cognitive dysfunction in the pediatric population of T1D.

### 4.2. The Impact of Dysregulated Insulin Signaling

Insulin signaling plays a crucial role in brain homeostasis too, influencing neuronal and synaptic function. Hypoinsulinemia in T1D, as well as hyperinsulinemia in T2D, are thought to be factors that can contribute to the brain’s metabolic changes and neuroinflammation affecting cognitive function and especially learning and memory skills, mainly through alterations in the activation of insulin receptors in the brain microstructure [87]. Several lines of evidence in the international literature reveal the impact of insulin deprivation in T1D on specific brain structures such as the glutamatergic synapses in hippocampal neurons, resulting in memory deficits [88]. The study of Creo et al. has shown that even transient insulin deprivation for few hours in patients with T1D can have a negative impact on cognitive function, reducing brain adenosine triphosphate (ATP) levels [89]. Especially, studies in adults with T2D and dementia have demonstrated that changes in tau protein and amyloid have been related to insulin resistance, leading to cognitive decline in these patients [90].

### 4.3. Blood–Brain Barrier (BBB) Impairment

Diabetes mellitus is related to blood–brain barrier disruption, which contributes to the appearance of complications regarding CNS function in patients with diabetes [91]. Appropriate BBB function is essential for the limited transport of molecules between the blood and brain, thus maintaining neuronal homeostasis. In circumstances of hyperglycemia, the occurring alterations of BBB integrity result in the disruption of molecular transport and, in many cases, increase its permeability [92]. Inflammatory processes, oxidative stress and production of AGEs take place in the BBB structure, influencing not only endothelial cells but also pericytes and astrocytes, leading to complications such as vascular dysfunction, cognitive deficits and dementia [93].

**Figure 1:**
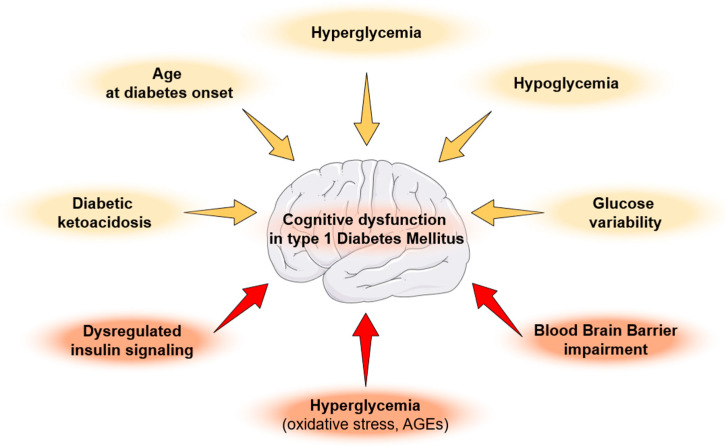
Risk factors (highlighted in light orange color) and molecular mechanisms (highlighted in red color) underlying cognitive dysfunction in children and adolescents with type 1 diabetes mellitus. AGEs: advanced glycation end products.

Figure 1 summarizes the above-mentioned risk factors and molecular mechanisms underlying cognitive dysfunction in children and adolescents with T1D.

### 4.4. Biomarkers Indicating Cognitive Impairment in Human and Animal Models

Several studies aim to identify biomarkers for the early diagnosis and management of cognitive impairment in patients with either type 1 or type 2 diabetes. Karger et al. demonstrated that adults with T1D enrolled in the DCCT/EDIC study exhibited higher plasma concentrations of neurofilament light chain (NFL) and phosphorylated Tau 181 (pTau-181) associated with lower psychomotor and mental efficiency, which were assessed through the appropriate cognitive tests. No association was found with their performance in tasks for memory testing. NFL and p Tau-181 are considered, among other proteins, such as amyloid β-40, amyloid- β-42 and glial fibrillary acidic protein (GFAP), as biomarkers related to brain damage. These proteins have also been linked to Alzheimer’s disease (AD), when expressed according to a typical amyloid positive profile. Nevertheless, in the above-mentioned study, no association was found between the expression of the amyloid proteins and glial fibrillary acidic protein (GFAP) to the cognitive impairment of patients with T1D, and neither was their amyloid proteomic pattern associated with the typical MRI findings in AD, such as AD signature region atrophy. As a result, the authors conclude that there is a profile of these biomarkers in their cohort of 373 adults with T1D, which is different to that in AD; thus, cognitive impairment in these patients probably takes place through non-amyloid mechanisms and should be distinct from AD [94]. Another study of Fonseca et al., including 114 adults with T1D, has also demonstrated an association between higher expression of plasma proteins, phosphorylated Tau 181 (pTau-181), and glial fibrillary acidic protein (GFAP), with lower performance in memory tasks. In disagreement with the DCCT/EDIC study, where no correlation was found, increased expression of the proteins β-amyloid 40 and β-amyloid42 were associated with a better vocabulary [95]. In large cohorts of patients with either T1D or T2D who were included in a recent meta-analysis, increased concentrations of myoinositol (MI) and decreased concentrations of Glu (glutamate), Glx (combined measurement of glutamate and glutamine) and NAA/Cr (N-acetyl aspartate/creatine) have been proposed as biomarkers for the detection of cognitive decline in patients with diabetes, especially those with T2D, at early stages of the disease. Simultaneously, it has been shown that the hippocampus is the brain structure that is more often affected by these metabolic alterations [96]. Decreased expression of glutathione in animal models was linked with increased oxidative stress in different brain regions, resulting in brain vulnerability to severe episodes of hypoglycemia. Indeed, when treatment with the antioxidant N-acetyl-L-cysteine (NAC) was administered in those animal models, a positive impact on neuronal survival and brain function occurred [97]. Yue et al. have recently indicated in a systematic review that CD3D, a T-cell surface regulatory molecule of immune check point, is a possible biomarker for early identification of mild cognitive impairment in T1D, which seemed to increase in animal models that started to experience deficits in cognitive function [98]. Finally, the role of high-mobility group box 1 (HMGB1), a protein activating NLRP3 inflammasome resulting in pyroptosis in vascular vessels, has been recently identified as a possible therapeutic biomarker for cognitive decline in diabetes mellitus [99]. To the best of our knowledge, so far there are no studies in children with T1D trying to identify biomarkers, such as markers of inflammation, neuroinflammation, complement activation and oxidative stress that have already been described in the literature to be related to T1D in children [100], that are possibly related to cognitive dysfunction in childhood.

## 5. Conclusions

Cognitive impairment in children with T1D, even when mild, is one of the less studied complications of the disease that can have a negative impact on a child’s mental health, influencing their ability to comply with their insulin regimen and achieve the recommended glycemic targets. Deficits in cognitive function become more prominent when diabetes is diagnosed before the age of 5 years and when diabetic ketoacidosis takes place at diabetes onset. As a result, it is of great importance for all health care systems worldwide to take measures for the early diagnosis of the disease to avoid the negative effects of DKA. Achieving good glycemic targets from early childhood is of paramount importance to minimize the deleterious effects on brain function of both protracted hyper- and hypoglycemia, as well as extreme glycemic variability. New technologies used in the monitoring and treatment of T1D, such as continuous glucose monitoring and automated insulin delivery systems, can further contribute to optimal glycemic control minimizing brain damage due to glycemic excursions in childhood. Large, prospective international studies using standardized cognitive testing methods to investigate the correlation between continuous glucose metrics and the results of cognitive testing are needed to identify all possible risk factors and biomarkers for the early detection of cognitive impairment in young children and adolescents with T1D.

## Figures and Tables

**Table 1:** Summary of relevant studies assessing DKA as a possible risk factor for cognitive impairment in children with T1D.

Study (Author/Year)	Sample Population	Study Design	Methods	Results	Limitations
Ghetti et al., 2020 [16]	*n*: 768 with T1D [*n*: 758 with DKA (*n*: 430 moderate/severe DKA, *n*: 328 mild DKA), *n*: 376 without DKA] Age: 6–18 years	Longitudinal (follow-up period of 6 months post-diagnosis)	Color tasks; WISC; Digit span recall	Negative impact of moderate/severe DKA on intelligence and memory. Negative impact of repeated DKA on intelligence	No control group
Semenkovich et al., 2016 [18]	*n*: 66 with T1D (*n*: 17 with DKA, *n*: 49 without DKA) *n*: 33 healthy controls Age: 7–17 years	Cross sectional	WJIII; CMS; SDR; Go-No-Go Task	Negative impact of DKA at diagnosis on memory 3.5 months after T1D onset	Small sample size of the DKA group
Cameron et al., 2014 [27]	*n*: 95 with T1D (*n*: 36 with DKA, *n*: 59 without DKA) Age: 6–18 years	Prospective Baseline time point: T1D onset Three follow-up time points within 6 months post-diagnosis	MRI; MRS; SYSTEMS; paired memory learning tasks; Test of Everyday Attention of Children	Negative impact of DKA at diagnosis on memory and attention 6 months after diagnosis, related to structural changes in total white volume reported 5 days after DKA at diabetes onset	Short follow-up period Only three-quarters of the participants completed follow-up No control group
Jessup et al., 2015 [31]	*n*: 11 with T1D (*n*: 4 with DKA, *n*: 7 without DKA) Age: 7–18 years	Longitudinal (follow-up period 8–12 weeks post-diagnosis)	MMSE; WRAML-2; Stanford–Binet; WISC-IV; DKEFS	Negative impact of DKA at diagnosis on visual cognitive skills	Small sample size No control group Short period of follow-up
Aye et al., 2019 [32]	*n*: 144 with T1D (*n*: 30 with moderate/severe DKA, *n*: 98 with mild DKA or without DKA) Age: 4–10 years	Prospective (follow-up period of 18 months)	MRI; WPPSI3/WASI; CPT2; NEPSYII; WJIII; CMS	Negative impact of the history of moderate/severe DKA on attention and IQ scoring Increased total white and gray matter volume when moderate/severe DKA has occurred	Small sample size of the moderate/severe DKA group No assessment of cognitive function at the time of DKA upon diagnosis
Ghetti et al., 2023 [33]	*n*: 73 with T1D (*n*: 46 with DKA, *n*: 27 without DKA) Age: 3–5 years	Cross sectional	WPPSI-3	Negative impact of DKA at diagnosis, regardless of the severity, on IQ performance	Small sample size No control group
Hannonen et al., 2012 [34]	*n*: 63 with T1D Age: 9–10 years *n* = 92 age-matched healthy controls	Cross sectional	WISC-III	No impact of DKA at diagnosis on academic performance	Small sample size Participants only with early diabetes onset
Ly et al., 2011 [35]	*n*: 33 with T1D Age: 6–15 years *n*: 34 age- and sex-matched controls	Longitudinal (10 year follow-up period)	WISC-IV; WMS-IV; CCFIT; WCST; YSR; BDI-II; STAI	No impact of DKA at diagnosis on memory, intelligence, executive function	42% of the participants participated in the follow-up assessment
He et al., 2018 [36]	*n* = 105 with T1D Age: 7–17 years *n* = 90 sex- and age-matched controls	Cross sectional	WISC-RC; WMS-RC	Negative impact of DKA at diagnosis on IQ scoring in children with T1D	Subgroup sample sizes relatively small
Al-Shehaili et al., 2023 [37]	*n*: 50 with T1D (*n*: 11 without DKA, *n*: 19 with mild DKA, *n*: 20 with moderate/severe DKA) Age: 5–14 years	Cross sectional	Stanford–Binet test	Negative impact of DKA at diagnosis on nonverbal tasks Negative impact of moderate/severe DKA on overall intelligence quotient	Modest sample size No control group
Schwartz et al., 2014 [38]	*n*: 147 with T1D (*n*: 47 with DKA) Age: 5–18 years	Prospective	GP; VMI; TMT; FAS; DS	No impact of DKA at diagnosis on neurocognitive testing of psychomotor speed one-year post-diagnosis	No control group

Abbreviations: DKA, diabetic ketoacidosis; T1D, type 1 diabetes; WISC, Wechsler Intelligence Scale for Children; WJIII, Woodcock–Johnson III; CMS, Children’s Memory Scale; SDR, Spatial Delayed Response; MRI, magnetic resonance imaging; MRS, magnetic resonance spectroscopy; SYSTEMS, Screening Test for the Evaluation of Mental Status; MMSE, Mini-Mental Status Exam; WRAML-2, Wide Range Assessment of Memory and Learning, Second Edition; DKEFS, Delis Kaplan Executive Function System; WPPSI3, Wechsler Preschool and Primary Scale of Intelligence, 3rd edition; WASI, Wechsler Adult Scale of Intelligence; CPT2, Conners’ Continuous Performance Test II; NEPSYII, neuropsychological assessment; WMS, Wechsler Memory Scale; CCFIT, Cattell Culture Fair Intelligence Test; WCST Wisconsin Card Sorting Test; YSR, Youth Self Report and Adult Self Report; BDI-II, Beck Depression Inventory; STAI, State-Trait Anxiety Index and State-Trait Anxiety Index for Children; GP, full-scale intelligence quotient Grooved Pegboard; VMI, visual–motor integration; TMT, Trail Making Test; FAS, Verbal Fluency Test; DS, Digit span.

**Table 2:** Summary of relevant studies assessing age at diagnosis as a possible risk factor of cognitive impairment in children with T1D.

Study (Author/Year)	Sample Population	Study Design	Methods	Results	Limitations
Mazaika et al., 2016 [42]	*n*: 141 with T1D diagnosed before 7 years Age: 4–10 years *n*: 69 age-matched controls	Longitudinal (18 months follow-up period)	MRI	Negative impact of younger age at T1D onset on growth rates of white and gray matter volume, in an age- dependent manner	Analysis of brain volume at each time point may be affected by hydration, long term glycemic control or diurnal glycemic or osmotic fluctuations
Hannonen et al., 2012 [34]	*n*: 63 with T1D diagnosed before 5 years Age: 9–10 years *n* = 92 age-matched healthy controls	Cross sectional	WISC-III	Negative impact of early-onset diabetes on spelling and mathematics	Small sample size Participants only with early onset of diabetes
Northam et al., 2001 [43]	*n*: 90 with T1D (*n*: 24 diagnosed before the age of 4 years) Age: 6–17 years *n*: 84 controls mean age: 12.1 years	Longitudinal (6 year follow-up period)	WISC-III; CNT; RAVLT; COWAT	Negative impact of early T1D onset (before the age of 4 years) on attention, executive function and processing speed of children with T1D	Not all participants completed the follow-up period of cognitive testing
Ferguson et al., 2005 [44]	*n*: 71 with T1D (*n* = 26 diagnosed before 7 years, *n* = 45 diagnosed after 7 years) Age: 20–44 years	Cross sectional	MRI; Hospital Anxiety and Depression Scale; WAIS-R; NART; CRT; Borkowski Verbal Fluency Test; PASAT	Negative impact of early T1D onset on nonverbal intelligence and information processing ability. Young adults with early onset of T1D exhibited mild brain atrophy	No control group No separate measurement of gray or white matter volume
Dogra et al., 2022 [47]	*n*: 73 adults with T1D (*n*: 23 diagnosed before 6 years, *n*: 25 diagnosed 7–12 years, *n*: 25 diagnosed 13–18 years) mean age: 24 years *n*: 27 healthy controls mean age: 27.6 years	Cross sectional	DS; SS; LNS; TMT-A&B; COWAT; AVLT; CFT; BVMGT; Digit Symbol Substitution Test	No impact of the age of T1D onset on attention, learning, memory and information processing speed	Not described
Northam et al., 2009 [45]	*n*: 106 with T1D (*n*: 38 diagnosed before 5 years) Mean age 20.5 years *n*: 75 controls, Mean age 21 years	Longitudinal (12 year follow-up period)	MRI; MRS; Wechsler Abbreviated Scale of General Intelligence	Early onset of T1D negatively related to IQ scoring and associated with alterations in brain structure	Not all participants completed the assessment in the follow-up period
Ly et al., 2011 [35]	*n*: 33 with T1D diagnosed before the age of 6 years Age: 6–15 years *n*: 34 age-, sex-matched controls	Longitudinal (10 year follow-up period)	WISC-IV; WMS-IV; CCFIT; WCST; YSR; BDI-II; STAI	No impact of early onset of T1D (before 6 years) on intelligence or memory	42% participated in the follow-up assessment
He et al., 2018 [36]	*n* = 105 with T1D (*n*: 24 diagnosed before 7 years, *n*: 81 diagnosed after 7 years) Age: 7–17 years *n* = 90 sex- and age-matched controls	Cross sectional study	WISC-RC; WMS-RC	Negative impact of early T1D onset on visuospatial perception ability and delayed logical memory	Subgroup sample sizes relatively small
Al-Shehaili et al., 2023 [37]	*n*: 50 with T1D (*n*: 15 diagnosed before 5 years) Age: 5–14 years	Cross sectional study	Stanford–Binet test	No impact of the age of T1D onset on cognitive performance	Modest sample size No control group
Schwartz et al., 2014 [38]	*n*: 147 with T1D aged 5–18 years (diagnosed *n*: 50, <8 years; *n*: 54, 9–11 years; *n*: 43, 12–18 years)	Prospective	GP; VMI; TMT; FAS; DS	No impact of the age of T1D onset on psychomotor speed one year post-diagnosis	No control group

Abbreviations: T1D, type 1 diabetes; MRI, magnetic resonance imaging; WISC, Wechsler Intelligence Scale for Children; CNT, Contingency Naming Test; RAVLT, Rey Auditory Verbal Learning Test; COWAT, Controlled Oral Word Association Test; WAIS-R, Wechsler Adult Intelligence Scale Revised; NART, National Adult Reading Test; CRT, choice reaction time; PASAT, Paced Auditory Serial Addition Task; DS, digit span; SS, spatial span; LNS, letter number sequencing, TMT-A&B, Trail Making Test—A&B; AVLT, Auditory Verbal Learning Test; CFT, Complex Figure Test; BVMGT, Bender Visual–Motor Gestalt Test; MRS, magnetic resonance spectroscopy; WMS, Wechsler Memory Scale; CCFIT, Cattell Culture Fair Intelligence Test; WCST Wisconsin Card Sorting Test; YSR, Youth Self Report and Adult Self Report; BDI-II, Beck Depression Inventory; STAI, State-Trait Anxiety Index and State-Trait Anxiety Index for Children; GP, full-scale intelligence quotient Grooved Pegboard; VMI, visual–motor integration; TMT, Trail Making Test; FAS, Verbal Fluency Test.

**Table 3:** Summary of relevant studies assessing hyperglycemia as a possible risk factor of cognitive impairment in children with T1D.

Study (Author/Year)	Sample Population	Study Design	Methods	Results	Limitations
Omladič et al., 2020 [17]	*n*: 20 with T1D Mean age 12.6 years *n*: 20 controls age-matched	Cross sectional	fMRI; MRS; sWM	Negative impact of acute hyperglycemia on lower spatial working capacity of children with T1D	Small sample size
Aye et al., 2019 [32]	*n*: 144 with T1D Age: 4–10 years	Prospective (follow-up period of 18 months)	MRI; WPPSI3/WASI; CPT2; NEPSYII; WJIII; CMS	No impact of hyperglycemia on cognitive performance	Small sample size of the moderate/severe DKA group
Mazaika et al., 2016 [42]	*n*: 141 with T1D Age: 4–10 years *n*: 69 controls age-matched	Longitudinal (18 months follow-up period)	MRI	Negative impact of hyperglycemia on mean curvature of cortical surface	Analysis of brain volume can be affected by hydration, long term glycemic control or diurnal glycemic or osmotic fluctuations
Hannonen et al., 2012 [34]	*n*: 63 with T1D and early onset of T1D Age: 9–10 years *n* = 92 age-matched controls	Cross sectional	WISC-III	Negative impact of high HbA1c one year after T1D diagnosis on spelling, independently of the mean or the recent HbA1c prior to testing	Small sample size Participants only with early onset of diabetes
Ly et al., 2011 [35]	*n*: 33 with T1D Age: 6–15 years *n*: 34 controls age-, sex-matched	Longitudinal (10 year follow-up period)	WISC-IV; WMS-IV; CCFIT; WCST; YSR; BDI-II; STAI	No impact of hyperglycemia on intelligence, memory, executive function	42% of the participants participated in the follow-up assessment
Davis et al., 1996 [49]	*n*: 12 with T1D Mean age: 12.4 years	Cross sectional	WISC-III	Lower IQ scoring in children with T1D and acute hyperglycemia compared to their scoring in conditions of euglycemia	No limitations are described
Winnick et al., 2017 [51]	*n*: 252 with T1D Mean age: 12.4 years	Longitudinal (Follow-up period of 2.5 years)	GPA	Better academic performance when the increase in HbA1c throughout the years was lower	No usage of a standardized achievement assessment tool
Stanislawska-Kubiak et al., 2018 [52]	*n* = 68 with T1D Age: 6–17 years stratified in 3 groups according to HbA1c level	Cross sectional	WISC-R; Trail of 10 words; Bricken Kamp’s and Zillmer’s d2 Test of Attention	Negative impact of higher HbA1c on verbal intelligence, no impact on attention and memory.	The group with the highest HbA1c (>8.6%) was the largest and oldest
He et al., 2018 [36]	*n* = 105 with T1D (*n*: 32 with good glycemic control, *n*: 73 with poor glycemic control) Age: 7–17 years *n* = 90 sex- and age-matched controls	Cross sectional	WISC-RC; WMS-RC	Negative impact of long periods in hyperglycemia on visuospatial perception ability within T1D group.	Subgroup sample sizes relatively small
Al-Shehaili et al., 2023 [37]	*n*: 50 with T1D Age: 5–14 years	Cross sectional	Stanford–Binet test	Lower scoring in verbal intelligence scales and working memory testing in children with T1D and poor glycemic control	Modest sample size No control group Lack of questionnaires available in Arabic language
Mauras et al., 2021 [28]	*n*: 144 with T1D *n*: 72 age-matched controls Mean age: 7 ± 1.7 years	Longitudinal (12 year follow-up period)	MRI; WISC	Negative impact of higher HbA1c on total brain volume and IQ scoring in children with T1D	26% of the initial cohort needed to be replaced after the 6 years follow-up period

Abbreviations: T1D, type 1 diabetes; fMRI, functional magnetic resonance imaging; MRS, magnetic resonance spectroscopy; sWM, spatial working memory task; MRI, magnetic resonance imaging; WPPSI3, Wechsler Preschool and Primary Scale of Intelligence, 3rd edition; WASI, Wechsler Adult Scale of Intelligence; CPT2, Conners’ Continuous Performance Test II; NEPSYII, neuropsychological assessment; WJIII, Woodcock–Johnson III; CMS, Children’s Memory Scale; WISC, Wechsler Intelligence Scale for Children; WMS, Wechsler Memory Scale; CCFIT, Cattell Culture Fair Intelligence Test; WCST, Wisconsin Card Sorting Test; YSR, Youth Self Report and Adult Self Report; BDI-II, Beck Depression Inventory; STAI, State-Trait Anxiety Index and State-Trait Anxiety Index for Children; and GPA, grade point average.

**Table 4:** Summary of relevant studies assessing hypoglycemia as a possible risk factor for cognitive impairment in children with T1D.

Study (Author/Year)	Sample Population	Study Design	Methods	Results	Limitations
Hannonen et al., 2012 [34]	*n*: 63 with T1D Age: 9–10 years *n* = 92 controls age-matched	Cross sectional	WISC-III	Younger age at a severe hypoglycemic event has a negative impact on the performance in mathematics	Small sample size Participants only with early onset of diabetes
Northam et al., 2009 [45]	*n*: 106 with T1D Mean age 20.5 years *n*: 75 controls Mean age 21 years	Longitudinal (12 year follow-up period)	MRI; MRS; Wechsler Abbreviated Scale of General Intelligence	Negative impact of SH on verbal and overall intelligence	Not all participants completed the assessment in the follow-up period
Ly et al., 2011 [35]	*n*: 33 with T1D Age: 6–15 years *n*: 34 age-, sex-matched controls	Longitudinal (10 year follow-up period)	WISC-IV; WMS-IV; CCFIT; WCST; YSR; BDI-II; STAI	Hypoglycemia had no impact on intelligence, memory and executive function	42% participated in the follow-up assessment
Al-Shehaili et al., 2023 [37]	*n*: 50 with T1D Age: 5–14 years	Cross sectional	Stanford–Binet test	Negative impact of frequent hypoglycemia on verbal knowledge Impact of SH on verbal and nonverbal fluid reasoning, knowledge, working memory	Modest sample size No control group
Musen et al., 2008 [62]	*n*: 82 with T1D on ICT *n*: 93 with T1D on ST Age: 13–19 years	Longitudinal (18 years follow-up period)	WAIS; HRNB; WMS; Digit Vigilance Test, GP; Verbal Fluency Test; Four-Word Short-Term Memory Test; Symbol-Digit Learning Test; Embedded Figures Test	Higher rates of SH were not associated with decline in any cognitive domain tested	Only adolescents included Relatively small sample size
Hershey et al., 2005 [63]	*n*: 103 with T1D Age 6–18 years *n*: 60 controls Age: 6–16 years	Retrospective	SDR	More than 3 episodes of SH, especially before the age of five, were associated with deficits in memory	Possible unreported or unrecognized episodes of SH that were not have been reported
He et al., 2018 [64]	*n*: 1355 with T1D *n*: 696 controls Age: 4–18 years	Meta-Analysis	Cognitive tasks evaluating intelligence, memory, attention, executive function, psychomotor speed	Negative impact of early SH (before the age of 7 years) on overall cognition compared to late SH	Large heterogeneity of the samples and cognitive tests being used
Aye et al., 2011 [65]	*n*: 27 with T1D Mean age 7 years *n*: 17 controls age-matched	Cross sectional	WPPSI; WISC; NEPSY; MRI	Lower WISC scores and gray and white matter volume in children with T1D who had experienced SH	Larger cross sectional and longitudinal studies are needed

Abbreviations: T1D, type 1 diabetes; WISC, Wechsler Intelligence Scale for Children; MRI, magnetic resonance imaging; MRS, magnetic resonance spectroscopy; SH, severe hypoglycemia; WMS, Wechsler Memory Scale; CCFIT, Cattell Culture Fair Intelligence Test; WCST Wisconsin Card Sorting Test; YSR, Youth Self Report and Adult Self Report; BDI-II, Beck Depression Inventory; STAI, State-Trait Anxiety Index and State-Trait Anxiety Index for Children; WAIS, Wechsler Adult Scale of Intelligence Digit Vigilance Test; HRNB, Halstead–Reitan Neuropsychological Test Battery; GP, Grooved Pegboard Test; SDR, spatial delayed response task; WPPSI, Wechsler Preschool and Primary Scale of Intelligence; NEPSY, A developmental neuropsychological assessment.

## Data Availability

No new data were created or analyzed in this study.

## Appendix A

**Table A1:** Risk of bias assessment of the selected studies, using the Newcastle–Ottawa Quality Assessment Scale ^a^.

Author/Year	Selection				Comparability		Outcome			Total
	Representativeness of the Exposed Cohort	Selection of the Non Exposed Cohort	Ascertainment of Exposure	Demonstration that Outcome Not Present at Start	Comparability of Cohorts (Age)	Comparability of Cohorts (Other Factors) ^b^	Ascertainment of Outcome	Enough Follow-Up for Outcomes to Occur ^c^	Adequacy of Follow-Up of Cohorts	
Ghetti et al., 2020 [16]	*	*	*	-	*	*	*	*	*	8
Omladič et al., 2020 [17]	*	*	*	*	*	-	*	*	*	8
Semenkovich et al., 2016 [18]	*	*	*	-	*	*	*	-	*	7
Cameron et al., 2014 [27]	*	*	*	-	*	*	*	*	-	7
Mauras et al., 2021 [28]	*	*	*	*	*	*	*	*	*	9
Jessup et al., 2015 [31]	*	*	*	-	*	-	*	*	*	7
Aye et al., 2019 [32]	*	*	*	-	*	*	*	*	-	7
Ghetti et al., 2023 [33]	*	*	*	-	*	*	*	*	-	7
Mazaika et al., 2016 [42]	*	*	*	-	*	-	*	*	*	7
Hannonen et al., 2012 [34]	*	-	*	-	*	*	*	*	-	6
Northam et al., 2001 [43]	*	*	*	*	*	*	-	*	*	8
Ferguson et al., 2005 [44]	*	*	*	-	*	*	*	*	*	8
Dogra et al., 2022 [47]	*	*	*	-	*	*	*	*	*	8
Ly et al., 2011 [35]	*	-	*	*	*	*	*	*	-	7
Davis et al., 1996 [49]	*	-	*	*	*	-	*	*	-	6
Winnick et al., 2017 [51]	*	-	*	*	*	*	-	*	*	7
Stanislawska-Kubiak et al., 2018 [52]	*	*	*	-	*	-	*	-	-	5
He et al., 2018 [36]	*	-	*	-	*	*	*	*	*	7
Schwartz et al., 2014 [38]	*	-	*	-	*	*	*	*	*	7
Musen et al., 2008 [62]	*	-	*	*	*	*	*	*	*	8
Hershey et al., 2005 [63]	*	*	-	-	*	*	*	*	*	7
Aye et al., 2011 [65]	*	*	*	-	*	*	*	-	*	7

^a^ The Newcastle–Ottawa Scale (NOS) was used to assess the risk of bias in cohort studies. Studies were evaluated across three domains: selection (4 points), comparability (2 points), and outcome (3 points), with a maximum score of 9 points. Studies scoring 7–9 were considered low risk of bias, 5–6 moderate risk, and ≤4 high risk; 19 studies exhibited a low risk of bias and 3 studies exhibited moderate risk. ^b^ Comparability*:* Studies were awarded one star if adjusted for age and an additional star if adjusted for other important confounders (e.g., diabetes duration, HbA1c, socioeconomic status). ^c^ Adequate follow-up was defined as ≥6 months.

## References

[1] Libman I., Haynes A., Lyons S., Pradeep P., Rwagasor E., Tung J.Y., Jefferies C.A., Oram R.A., Dabelea D., Craig M.E. (2022). ISPAD Clinical Practice Consensus Guidelines 2022: Definition, epidemiology, and classification of diabetes in children and adolescents. Pediatr. Diabetes.

[2] Skyler J.S., Bakris G.L., Bonifacio E., Darsow T., Eckel R.H., Groop L., Groop P.-H., Handelsman Y., Insel R.A., Mathieu C. (2017). Differentiation of Diabetes by Pathophysiology, Natural History, and Prognosis. Diabetes.

[3] Rewers M., Ludvigsson J. (2016). Environmental risk factors for type 1 diabetes. Lancet.

[4] Kordonouri O., Cuthbertson D., Belteky M., Aschemeier-Fuchs B., White N.H., Cummings E., Knip M., Ludvigsson J. (2022). Infections in the first year of life and development of beta cell autoimmunity and clinical type 1 diabetes in high-risk individuals: The TRIGR cohort. Diabetologia.

[5] Bluestone J.A., Herold K., Eisenbarth G. (2010). Genetics, pathogenesis and clinical interventions in type 1 diabetes. Nature.

[6] Cengiz E., Danne T., Ahmad T., Ayyavoo A., Beran D., Codner E., Ehtisham S., Jarosz-Chobot P., Mungai L.N., Ng S.M. (2024). International Society for Pediatric and Adolescent Diabetes Clinical Practice Consensus Guidelines 2024: Insulin and Adjunctive Treatments in Children and Adolescents with Diabetes. Horm. Res. Paediatr..

[7] White N.H., Cleary P.A., Dahms W., Goldstein D., Malone J., Tamborlane W.V. (2001). Beneficial effects of intensive therapy of diabetes during adolescence: Outcomes after the conclusion of the Diabetes Control and Complications Trial (DCCT). J. Pediatr..

[8] de Bock M., Agwu J.C., Deabreu M., Dovc K., Maahs D.M., Marcovecchio M.L., Mahmud F.H., Nóvoa-Medina Y., Priyambada L., Smart C.E. (2024). International Society for Pediatric and Adolescent Diabetes Clinical Practice Consensus Guidelines 2024: Glycemic Targets. Horm. Res. Paediatr..

[9] Abraham M.B., Karges B., Dovc K., Naranjo D., Arbelaez A.M., Mbogo J., Javelikar G., Jones T.W., Mahmud F.H. (2022). ISPAD Clinical Practice Consensus Guidelines 2022: Assessment and management of hypoglycemia in children and adolescents with diabetes. Pediatr. Diabetes.

[10] Glaser N., Fritsch M., Priyambada L., Rewers A., Cherubini V., Estrada S., Wolfsdorf J.I., Codner E. (2022). ISPAD clinical practice consensus guidelines 2022: Diabetic ketoacidosis and hyperglycemic hyperosmolar state. Pediatr. Diabetes.

[11] Bjornstad P., Dart A., Donaghue K.C., Dost A., Feldman E.L., Tan G.S., Wadwa R.P., Zabeen B., Marcovecchio M.L. (2022). ISPAD Clinical Practice Consensus Guidelines 2022: Microvascular and macrovascular complications in children and adolescents with diabetes. Pediatr. Diabetes.

[12] Sjöholm Å., Bennet L., Nilsson P.M. (2025). Cognitive dysfunction in diabetes—The ‘forgotten’ diabetes complication: A narrative review. Scand. J. Prim. Health Care.

[13] Cameron F.J., Northam E.A., Ryan C.M. (2019). The effect of type 1 diabetes on the developing brain. Lancet Child Adolesc. Health.

[14] van Duinkerken E., Ryan C.M. (2020). Diabetes mellitus in the young and the old: Effects on cognitive functioning across the life span. Neurobiol. Dis..

[15] Dahlquist G., Källén B. (2007). Swedish Childhood Diabetes Study Group. School performance in children with type 1 diabetes—A population-based register study. Diabetologia.

[16] Ghetti S., Kuppermann N., Rewers A., Myers S.R., Schunk J.E., Stoner M.J., Garro A., Quayle K.S., Brown K.M., Trainor J.L. (2020). Cognitive Function Following Diabetic Ketoacidosis in Children with New-Onset or Previously Diagnosed Type 1 Diabetes. Diabetes Care.

[17] Omladič J.Š., Ozimič A.S., Vovk A., Šuput D., Repovš G., Dovc K., Bratina N., Stefanija M.A., Battelino T. (2020). Acute Hyperglycemia and Spatial Working Memory in Adolescents With Type 1 Diabetes. Diabetes Care.

[18] Semenkovich K., Bischoffb A., Dotyb T., Nelsona S., Sillerc A.F., Hershey T., Arbelaez A.M. (2016). Clinical presentation and memory function in youth with type 1 diabetes. Pediatr. Diabetes.

[19] Gallardo-Moreno G.B., González-Garrido A.A., Villaseñor-Cabrera T., Alvarado-Rodríguez F.J., Ruiz-Stovel V.D., Jiménez-Maldonado M.E., Contreras-Piña N., Gómez-Velázquez F.R. (2020). Sustained attention in schoolchildren with type-1 diabetes. A quantitative EEG study. Clin. Neurophysiol..

[20] Lancrei H.M., Yeshayahu Y., Grossman E.S., Berger I. (2022). Sweet but sour: Impaired attention functioning in children with type 1 diabetes mellitus. Front. Hum. Neurosci..

[21] He J., Zhu J., Xie Y., Du H., Li S., Li S., He W., Li X., Zhou Z., Zhu X. (2020). Effects of Diabetic Ketoacidosis on Executive Function in Children With Type 1 Diabetes: Evidence From Wisconsin Card Sorting Test Performance. Psychosom. Med..

[22] Swaminathan K., Nanda P.M., Yadav J., Malhi P., Kumar R., Sharma A., Sharma R., Dayal D. (2025). Cognitive Function in Early Onset Type 1 Diabetes in Children. Indian J. Pediatr..

[23] Goethals E.R., Lemiere J., Snoek F.J., Casteels K., Luyckx K., Wit M. (2021). Executive function mediates the link between externalizing behavior and HbA1c in children and adolescents with type 1 diabetes: A cross-national investigation. Pediatr. Diabetes.

[24] Shalimova A., Graff B., Gąsecki D., Wolf J., Sabisz A., Szurowska E., Jodzio K., Narkiewicz K. (2019). Cognitive Dysfunction in Type 1 Diabetes Mellitus. J. Clin. Endocrinol. Metab..

[25] Song J.-W., Huang X.-Y., Huang M., Cui S.-H., Zhou Y.-J., Liu X.-Z., Yan Z.-H., Ye X.-J., Liu K. (2024). Abnormalities in spontaneous brain activity and functional connectivity are associated with cognitive impairments in children with type 1 diabetes mellitus. J. Neuroradiol..

[26] Stanisławska-Kubiak M., Majewska K.A., Krasińska A., Wais P., Majewski D., Mojs E., Kȩdzia A. (2024). Brain functional and structural changes in diabetic children. How can intellectual development be optimized in type 1 diabetes?. Ther. Adv. Chronic Dis..

[27] Cameron F.J., Scratch S.E., Nadebaum C., Northam E.A., Koves I., Jennings J., Finney K., Neil J.J., Wellard R.M., Mackay M. (2014). DKA Brain Injury Study Group. Neurological consequences of diabetic ketoacidosis at initial presentation of type 1 diabetes in a prospective cohort study of children. Diabetes Care.

[28] Mauras N., Buckingham B., White N.H., Tsalikian E., Weinzimer S.A., Jo B., Cato A., Fox L.A., Aye T., Arbelaez A.M. (2021). For the Diabetes Research in Children Network (DirecNet)*. Impact of Type 1 Diabetes in the Developing Brain in Children: A Longitudinal Study. Diabetes Care.

[29] Toprak H., Yetis H., Alkan A., Filiz M., Kurtcan S., Aralasmak A., Aksu M.Ş., Cesur Y. (2016). Relationships of DTI findings with neurocognitive dysfunction in children with Type 1 diabetes mellitus. Br. J. Radiol..

[30] O’cOnnell M.A., Beare R., Messazos B., Northam E.A., Fletcher M.C., Seal M.L., Cameron F.J. (2025). Acute brain responses to hypoglycaemia and hyperglycaemia in adolescents with type 1 diabetes. Diabetologia.

[31] Jessup A.B., Grimley M.B., Meyer E., Passmore G.P., Belger A., Hoffman W.H., Çalıkoğlu A.S. (2015). Effects of Diabetic Ketoacidosis on Visual and Verbal Neurocognitive Function in Young Patients Presenting with New-Onset Type 1 Diabetes. J. Clin. Res. Pediatr. Endocrinol..

[32] Aye T., Mazaika P.K., Mauras N., Marzelli M.J., Shen H., Hershey T., Cato A., Weinzimer S.A., White N.H., Tsalikian E. (2019). Impact of Early Diabetic Ketoacidosis on the Developing Brain. Diabetes Care.

[33] Ghetti S., Kuppermann N., Rewers A., Myers S.R., Schunk J.E., Stoner M.J., Garro A., Quayle K.S., Brown K.M., Trainor J.L. (2023). Cognitive function following diabetic ketoacidosis in young children with type 1 diabetes. Endocrinol. Diabetes Metab..

[34] Hannonen R., Komulainen J., Riikonen R., Ahonen T., Eklund K., Tolvanen A., Keskinen P., Nuuja A. (2012). Academic skills in children with early-onset type 1 diabetes: The effects of diabetes-related risk factors. Dev. Med. Child Neurol..

[35] Ly T.T., Anderson M., McNamara K.A., Davis E.A., Jones T.W. (2011). Neurocognitive outcomes in young adults with early-onset type 1 diabetes: A prospective follow-up study. Diabetes Care.

[36] He J., Li S., Liu F., Zheng H., Yan X., Xie Y., Li X., Zhou Z., Zhu X. (2018). Glycemic control is related to cognitive dysfunction in Chinese children with type 1 diabetes mellitus. J. Diabetes.

[37] Al-Shehaili S.M., Al-Johani S.S., Al-Sarhan N.T., Al-Anazi A.A., Al-Mijmaj F.F., Al-Qhatani W.N., Al-Nasser L.M., Al-Yami D.R., Al-Razooq A.S. (2023). The effect of poor glycemic control on cognitive function in children and adolescents with type 1 diabetes mellitus: A single-center cross-sectional study (2019–2020). Saudi Med. J..

[38] Schwartz D.D., Axelrad M.E., Anderson B.J. (2014). Neurocognitive functioning in children and adolescents at the time of type 1 diabetes diagnosis: Associations with glycemic control 1 year after diagnosis. Diabetes Care.

[39] Waernbaum I., Lind T., Möllsten A., Dahlquist G. (2023). The incidence of childhood-onset type 1 diabetes, time trends and association with the population composition in Sweden: A 40-year follow-up. Diabetologia.

[40] Patterson C.C., Harjutsalo V., Rosenbauer J., Neu A., Cinek O., Skrivarhaug T., Rami-Merhar B., Soltesz G., Svensson J., Parslow R.C. (2019). Trends and cyclical variation in the incidence of childhood type 1 diabetes in 26 European centres in the 25 year period 1989–2013: A multicentre prospective registration study. Diabetologia.

[41] Tierney A.L., Nelson C.A. (2009). Brain Development and the Role of Experience in the Early Years. Zero Three.

[42] Mazaika P.K., Weinzimer S.A., Mauras N., Buckingham B., White N.H., Tsalikian E., Hershey T., Cato A., Aye T., Fox L. (2016). Diabetes Research in Children Network (DirecNet). Variations in Brain Volume and Growth in Young Children With Type 1 Diabetes. Diabetes.

[43] Northam E.A., Anderson P.J., Jacobs R., Hughes M., Warne G.L., Werther G.A. (2001). Neuropsychological profiles of children with type 1 diabetes 6 years after disease onset. Diabetes Care.

[44] Ferguson S.C., Blane A., Wardlaw J., Frier B.M., Perros P., McCrimmon R.J., Deary I.J. (2005). Influence of an early-onset age of type 1 diabetes on cerebral structure and cognitive function. Diabetes Care.

[45] Northam E.A., Rankins D., Lin A., Wellard R.M., Pell G.S., Finch S.J., Werther G.A., Cameron F.J. (2009). Central nervous system function in youth with type 1 diabetes 12 years after disease onset. Diabetes Care.

[46] Ryan C., Vega A., Drash A. (1985). Cognitive deficits in adolescents who developed diabetes early in life. Pediatrics.

[47] Dogra V., Mittal B., Kumaran S.S., Nehra A., Sagar R., Gupta A., Kalaivani M., Gupta Y., Tandon N. (2022). Evaluation of Cognitive Deficits in Adults with Type 1 Diabetes Stratified by the Age of Diabetes Onset: A Cross-Sectional Study. Adv. Ther..

[48] Hua W., Du Z., Lu T., Tian L. (2024). Effect of glycemic control on cognitive function in patients with type 1 diabetes mellitus: A systematic review and meta-analysis. Syst. Rev..

[49] Davis E.A., Soong S.-A., Byrne G.C., Jones T.W. (1996). Acute hyperglycaemia impairs cognitive function in children with IDDM. J. Pediatr. Endocrinol. Metab..

[50] Mauras N., Ma Q., Weinzimer S.A., White N.H., Tsalikian E., Buckingham B., Fox L.A., Tamborlane W., Arbelaez A.M., Tansey M. (2025). Diabetes Research in Children Network (DirecNet). Differences in White Matter Microstructure in Children With Type 1 Diabetes Persist During Longitudinal Follow-up: Relation to Dysglycemia. Diabetes.

[51] Winnick J.B., Berg C.A., Wiebe D.J., Schaefer B.A., Lei P.-W., Butner J.E. (2017). Metabolic control and academic achievement over time among adolescents with type 1 diabetes. Sch. Psychol. Q..

[52] Stanislawska-Kubiak M., Mojs E., Wojciak R.W., Piasecki B., Matecka M., Sokalski J., Kopczynski P., Fichna P. (2018). An analysis of cognitive functioning of children and youth with type 1 diabetes (T1DM) in the context of glycemic control. Eur. Rev. Med. Pharmacol. Sci..

[53] Nunley K.A., Rosano C., Ryan C.M., Jennings J.R., Aizenstein H.J., Zgibor J.C., Costacou T., Boudreau R.M., Miller R., Orchard T.J. (2015). Clinically Relevant Cognitive Impairment in Middle-Aged Adults With Childhood-Onset Type 1 Diabetes. Diabetes Care.

[54] Zaitoon H., Perl L., Cohen-Sela E., Oren A., Lebenthal Y., Brener A. (2026). The virtual Morris water maze for cognitive function assessment in adolescents with type 1 diabetes. Diabetologia.

[55] Languren G., Montiel T., Julio-Amilpas A., Massieu L. (2013). Neuronal damage and cognitive impairment associated with hypoglycemia: An integrated view. Neurochem. Int..

[56] Nevo-Shenker M., Shalitin S. (2021). The Impact of Hypo- and Hyperglycemia on Cognition and Brain Development in Young Children with Type 1 Diabetes. Horm. Res. Paediatr..

[57] Whitmer R.A., Gilsanz P., Quesenberry C.P., Karter A.J., Lacy M.E. (2021). Association of Type 1 Diabetes and Hypoglycemic and Hyperglycemic Events and Risk of Dementia. Neurology.

[58] Alsharif A.A., Wong I.C.K., Ma T., Lau W., Alhamed M., Alwafi H., Wei L. (2023). The association between dementia and the risk of hypoglycaemia events among patients with diabetes mellitus: A propensity-score matched cohort analysis. Front. Med..

[59] Mu Z., Sun M., Wen L., Li P., Gao J., Liu M., Bian H., Wang Z. (2024). Effect of hypoglycemia on cognitive performance in older patients with diabetes: A meta-analysis. Ann. Endocrinol..

[60] Reichard P., Pihl M., Rosenqvist U., Sule J. (1996). Complications in IDDM are caused by elevated blood glucose level: The Stockholm Diabetes Intervention Study (SDIS) at 10-year follow up. Diabetologia.

[61] Jacobson A.M., Musen G., Ryan C.M., Silvers N., Cleary P., Waberski B., Burwood A., Weinger K., Bayless M., Dahms W. (2007). Long-term effect of diabetes and its treatment on cognitive function. N. Engl. J. Med..

[62] Musen G., Jacobson A.M., Ryan C.M., Cleary P.A., Waberski B.H., Weinger K., Dahms W., Bayless M., Silvers N., Harth J. (2008). Impact of diabetes and its treatment on cognitive function among adolescents who participated in the Diabetes Control and Complications Trial. Diabetes Care.

[63] Hershey T., Perantie D.C., Warren S.L., Zimmerman E.C., Sadler M., White N.H. (2005). Frequency and timing of severe hypoglycemia affects spatial memory in children with type 1 diabetes. Diabetes Care.

[64] He J., Ryder A.G., Li S., Liu W., Zhu X. (2018). Glycemic extremes are related to cognitive dysfunction in children with type 1 diabetes: A meta-analysis. J. Diabetes Investig..

[65] Aye T., Reiss A.L., Kesler S., Hoang S., Drobny J., Park Y., Schleifer K., Baumgartner H., Wilson D.M., Buckingham B.A. (2011). The feasibility of detecting neuropsychologic and neuroanatomic effects of type 1 diabetes in young children. Diabetes Care.

[66] Sherr J.L., Laffel L.M., Liu J., Wolf W., Bispham J., Chapman K.S., Finan D., Titievsky L., Liu T., Hagan K. (2024). Severe Hypoglycemia and Impaired Awareness of Hypoglycemia Persist in People With Type 1 Diabetes Despite Use of Diabetes Technology: Results From a Cross-sectional Survey. Diabetes Care.

[67] Verhulst C.E., Fabricius T.W., Nefs G., Kessels R.P., Pouwer F., Teerenstra S., Tack C.J., Broadley M.M., Kristensen P.L., McCrimmon R.J. (2022). Consistent Effects of Hypoglycemia on Cognitive Function in People With or Without Diabetes. Diabetes Care.

[68] Watt C., Sanchez-Rangel E., Hwang J.J. (2020). Glycemic Variability and CNS Inflammation: Reviewing the Connection. Nutrients.

[69] Wang X., Cao Y. (2025). A Narrative Review: Relationship Between Glycemic Variability and Emerging Complications of Diabetes Mellitus. Biomolecules.

[70] Gonzales P.N.G., Ampil E.R., Rosa J.-A.S.C.-D., Villaraza S.G., Joson M.L.C. (2024). Increased Risk of Alzheimer’s Disease With Glycemic Variability: A Systematic Review and Meta-Analysis. Cureus.

[71] Chi H., Song M., Zhang J., Zhou J., Liu D. (2023). Relationship between acute glucose variability and cognitive decline in type 2 diabetes: A systematic review and meta-analysis. PLoS ONE.

[72] Hawks Z.W., Beck E.D., Jung L., Fonseca L.M., Sliwinski M.J., Weinstock R.S., Grinspoon E., Xu I., Strong R.W., Singh S. (2024). Dynamic associations between glucose and ecological momentary cognition in Type 1 Diabetes. npj Digit. Med..

[73] Meng X., Gong C., Cao B., Peng X., Wu D., Gu Y., Wei L., Liang X., Liu M., Li W. (2015). Glucose fluctuations in association with oxidative stress among children with T1DM: Comparison of different phases. J. Clin. Endocrinol. Metab..

[74] DeSalvo D.J., Miller K.M., Hermann J.M., Maahs D.M., Hofer S.E., Clements M.A., Lilienthal E., Sherr J.L., Tauschmann M., Holl R.W. (2018). T1D Exchange and DPV Registries. Continuous glucose monitoring and glycemic control among youth with type 1 diabetes: International comparison from the T1D Exchange and DPV Initiative. Pediatr. Diabetes.

[75] Gerhardsson P., Schwandt A., Witsch M., Kordonouri O., Svensson J., Forsander G., Battelino T., Veeze H.J., Danne T. (2021). The SWEET Project 10-Year Benchmarking in 19 Countries Worldwide Is Associated with Improved HbA1c and Increased Use of Diabetes Technology in Youth with Type 1 Diabetes. Diabetes Technol. Ther..

[76] Biester T., Berget C., Boughton C., Cudizio L., Ekhlaspour L., Hilliard M.E., Reddy L., Um S.S.N., Schoelwer M., Sherr J.L. (2024). International Society for Pediatric and Adolescent Diabetes Clinical Practice Consensus Guidelines 2024: Diabetes Technologies—Insulin Delivery. Horm. Res. Paediatr..

[77] Beato-Víbora P.I., Chico A., Moreno-Fernandez J., Bellido-Castañeda V., Nattero-Chávez L., Picón-César M.J., Martínez-Brocca M.A., Giménez-Álvarez M., Aguilera-Hurtado E., Climent-Biescas E. (2024). A Multicenter Prospective Evaluation of the Benefits of Two Advanced Hybrid Closed-Loop Systems in Glucose Control and Patient-Reported Outcomes in a Real-world Setting. Diabetes Care.

[78] Reiss A.L., Jo B., Arbelaez A.M., Tsalikian E., Buckingham B., Weinzimer S.A., Fox L.A., Cato A., White N.H., Tansey M. (2022). A Pilot randomized trial to examine effects of a hybrid closed-loop insulin delivery system on neurodevelopmental and cognitive outcomes in adolescents with type 1 diabetes. Nat. Commun..

[79] Hamed S.A. (2017). Brain injury with diabetes mellitus: Evidence, mechanisms and treatment implications. Expert Rev. Clin. Pharmacol..

[80] Yu X., He H., Wen J., Xu X., Ruan Z., Hu R., Wang F., Ju H. (2025). Diabetes-related cognitive impairment: Mechanisms, symptoms, and treatments. Open Med..

[81] Negre-Salvayre A., Salvayre R., Augé N., Pamplona R., Portero-Otín M. (2009). Hyperglycemia and glycation in diabetic complications. Antioxid. Redox Signal..

[82] Gupta M., Pandey S., Rumman M., Singh B., Mahdi A.A. (2022). Molecular mechanisms underlying hyperglycemia associated cognitive decline. IBRO Neurosci. Rep..

[83] Pan D., Xu L., Guo M. (2022). The role of protein kinase C in diabetic microvascular complications. Front. Endocrinol..

[84] Bornemann E.A., Kamma H.K., Alabbas M., Abid N., Manaye S., Cheran K., Murthy C., Giva S., Penumetcha S.S., Elashahab M. (2025). The Effect of Chronic Inflammation and Oxidative Stress on Alzheimer’s Disease Progression: A Systematic Review. Cureus.

[85] Amorim M.D.S.D.N., Rates E.R.D., Vitoria d.A.C.M.I., Filho J.F.S.D., dos Santos C.C., Santos-Oliveira R., Gaspar R.S., Sanches J.R., Pinto B.A.S., Paes A.M.d.A. (2024). Diabetes and Cognitive Decline: An Innovative Approach to Analyzing the Biophysical and Vibrational Properties of the Hippocampus. ACS Omega.

[86] Foland-Ross L.C., Reiss A.L., Mazaika P.K., Mauras N., Weinzimer S.A., Aye T., Tansey M.J., White N.H. (2018). Diabetes Research in Children Network (DirecNet). Longitudinal assessment of hippocampus structure in children with type 1 diabetes. Pediatr. Diabetes.

[87] Park C.R. (2001). Cognitive effects of insulin in the central nervous system. Neurosci. Biobehav. Rev..

[88] Shypshyna M., Kolesnyk O., Fedulova S., Veselovsky N. (2023). Insulin modulates the paired-pulse plasticity at glutamatergic synapses of hippocampal neurons under hypoinsulinemia. Front. Cell. Neurosci..

[89] Creo A.L., Cortes T.M., Jo H.J., Huebner A.R., Dasari S., Tillema J.-M., Lteif A.N., Klaus K.A., Ruegsegger G.N., Kudva Y.C. (2021). Brain functions and cognition on transient insulin deprivation in type 1 diabetes. JCI Insight.

[90] van der Heide L.P., Ramakers G.M., Smidt M.P. (2006). Insulin signaling in the central nervous system: Learning to survive. Prog. Neurobiol..

[91] Prasad S., Sajja R.K., Naik P., Cucullo L. (2014). Diabetes Mellitus and Blood-Brain Barrier Dysfunction: An Overview. J. Pharmacovigil..

[92] Chen C., Xu X., Lu J., Xiang Y., Shi L., Liu D. (2025). Hyperglycemia-induced blood-brain barrier dysfunction: Mechanisms and therapeutic interventions. Microvasc. Res..

[93] Long C., Pu Y., Keng S., Tao J., Zhang B., Yue R. (2025). Novel Insights into the Mechanism and Treatment of Diabetes-Related Brain Complications: Focusing on the Blood-Brain Barrier Impairment. Aging Dis..

[94] Karger A.B., Nasrallah I.M., Braffett B.H., Luchsinger J.A., Ryan C.M., Bebu I., Arends V., Habes M., Gubitosi-Klug R.A., Chaytor N. (2024). Plasma Biomarkers of Brain Injury and Their Association With Brain MRI and Cognition in Type 1 Diabetes. Diabetes Care.

[95] Fonseca L.M., Beeri M.S., Hawks Z.W., Jung L., Cleveland M., Delgado N., Bulger J., Grinspoon E., Janess K., Sliwinski M.J. (2024). ATN blood biomarkers are related to digital cognitive assessment in type 1 diabetes. Alzheimer’s Dement.

[96] Chen M., Deng C., Chen P., Li A., Wu H., Ouyang F., Hu X., Liu J., Wang S., Tang D. (2024). Non-invasive metabolic biomarkers in initial cognitive impairment in patients with diabetes: A systematic review and meta-analysis. Diabetes Obes. Metab..

[97] Languren G., Montiel T., Ramírez-Lugo L., Balderas I., Sánchez-Chávez G., Sotres-Bayón F., Bermúdez-Rattoni F., Massieu L. (2019). Recurrent moderate hypoglycemia exacerbates oxidative damage and neuronal death leading to cognitive dysfunction after the hypoglycemic coma. J. Cereb. Blood Flow Metab..

[98] Yue D., Wang R., Zhao Y., Wu B., Li S., Zeng W., Wan S., Liu L., Dai Y., Shi Y. (2024). Investigating the molecular mechanisms between type 1 diabetes and mild cognitive impairment using bioinformatics analysis, with a focus on immune response. Int. Immunopharmacol..

[99] Liu L., Wang N., Kalionis B., Xia S., He Q. (2022). HMGB1 plays an important role in pyroptosis induced blood brain barrier breakdown in diabetes-associated cognitive decline. J. Neuroimmunol..

[100] Moulder R., Bhosale S.D., Lahesmaa R., Goodlett D.R. (2017). The progress and potential of proteomic biomarkers for type 1 diabetes in children. Expert Rev. Proteom..

